# Effects of stressful life-events on DNA methylation in panic disorder and major depressive disorder

**DOI:** 10.1186/s13148-022-01274-y

**Published:** 2022-04-27

**Authors:** Darina Czamara, Alexa Neufang, Roman Dieterle, Stella Iurato, Janine Arloth, Jade Martins, Marcus Ising, Elisabeth E. Binder, Angelika Erhardt

**Affiliations:** 1 Translational Department, Max Planck Institute for Psychiatry, Kraepelinstrasse 2+10, 80804 Munich, Germany; 2 Institute of Statistics, Faculty of Mathematics, Informatics and Statistics, Ludwig-Maximilians-University Munich, Munich, Germany; 3 Department of Psychiatry and Behavioral Sciences, School of Medicine, Emory University, Atlanta, GA USA; 4 Department of Psychiatry, Psychosomatics and Psychotherapy, Centre of Mental Health, Julius-Maximilians-University, Wuerzburg, Germany

**Keywords:** Panic disorder, Major depressive disorder, Stressful life events, EWAS

## Abstract

**Background:**

Panic disorder (PD) is characterized by recurrent panic attacks and higher affection of women as compared to men. The lifetime prevalence of PD is about 2–3% in the general population leading to tremendous distress and disability. Etiologically, genetic and environmental factors, such as stress, contribute to the onset and relapse of PD. In the present study, we investigated epigenome-wide DNA methylation (DNAm) in respond to a cumulative, stress-weighted life events score (wLE) in patients with PD and its boundary to major depressive disorder (MDD), frequently co-occurring with symptoms of PD.

**Methods:**

DNAm was assessed by the Illumina HumanMethylation450 BeadChip. In a meta-analytic approach, epigenome-wide DNAm changes in association with wLE were first analyzed in two PD cohorts (with a total sample size of 183 PD patients and 85 healthy controls) and lastly in 102 patients with MDD to identify possible overlapping and opposing effects of wLE on DNAm. Additionally, analysis of differentially methylated regions (DMRs) was conducted to identify regional clusters of association.

**Results:**

Two CpG-sites presented with *p*-values below 1 × 10^−05^ in PD: cg09738429 (*p* = 6.40 × 10^−06^, located in an intergenic shore region in next proximity of *PYROXD1*) and cg03341655 (*p* = 8.14 × 10^−06^, located in the exonic region of *GFOD2*). The association of DNAm at cg03341655 and wLE could be replicated in the independent MDD case sample indicating a diagnosis independent effect. Genes mapping to the top hits were significantly upregulated in brain and top hits have been implicated in the metabolic system. Additionally, two significant DMRs were identified for PD only on chromosome 10 and 18, including CpG-sites which have been reported to be associated with anxiety and other psychiatric phenotypes.

**Conclusion:**

This first DNAm analysis in PD reveals first evidence of small but significant DNAm changes in PD in association with cumulative stress-weighted life events. Most of the top associated CpG-sites are located in genes implicated in metabolic processes supporting the hypothesis that environmental stress contributes to health damaging changes by affecting a broad spectrum of systems in the body.

**Supplementary Information:**

The online version contains supplementary material available at 10.1186/s13148-022-01274-y.

## Background

Panic disorder (PD) is characterized by recurrent, unexpected panic attacks which are associated with extreme anxiety and fear levels and a wide range of further psychological and somatic symptoms, such as fear of dying, feeling of being out of control, heart racing palpitations, difficulties to breathe and tightness in the chest [1]. Affected individuals often experience concerns about future panic attacks, which leads to phobic avoidance and long-term negative changes in daily life functions as well as psychological distress [2]. As such, PD is often associated with agoraphobia, characterized by panic attacks in situations where patients feel trapped or are unable to escape [3]. The lifetime prevalence of PD is about 2–3% in the general population and women are affected as twice as high as men [4, 5]. The comorbidity with further psychiatric conditions is high, specifically with anxiety disorders or depression [6]. Despite the availability of treatment options for PD, such as medication and psychotherapy, more than one third of patients respond only partially, continuing to have sub-threshold panic symptoms, and a considerable proportion of affected individuals relapse later in life [7].

The etiology of PD is considered to be complex involving genetic and environmental factors and their interaction [8]. Approximately 30–40% of disease etiology are assigned to genetics, consisting of common and rare variations across the genome and suggested higher proportion of genetic contribution in those reporting familial aggregation and early disease onset [9]. Following this, environmental influences, and more specifically, mostly unique individual experiences, are etiologically highly relevant on shaping biological processes which lead to the risk for clinical symptomatology [10]. According to the latter twin study, the proportion of variance in liability for PD attributable to environment ranges to 0.7 (0.59 individual environment, 0.11 shared environment). As such, stress is one of the candidate environmental triggers associated with higher risk for PD and anxiety disorders in general [11]. Although not many studies are available on specific stressors predisposing to PD, some evidence shows that childhood adversities, more recent separation and loss events as well as long-lasting stressful conditions, are associated with panic pathology with odds ratios ranging between 1.39 and 2.52 indicating substantial effects [12].

Environmental influences can induce long-lasting alterations in neurobiological systems, e.g., mediated by epigenetic mechanisms [13]. Epigenetics describes gene regulatory processes without changing the original DNA sequence. These modifications can be time-stable, heritable and responsive to environmental influences [14]. One of the epigenetic mechanisms increasingly studied in psychiatric research is DNA methylation (DNAm), which occurs on cytosines through addition of a methyl-group [15]. In consequence, this process modulates gene expression by regulating the accessibility of transcription factors to their binding sites.

The epigenetic research in PD is only at an early stage [16]. Most studies investigated DNAm between PD patients and controls on categorical level focusing on candidate genes from the monoamine systems, but first epigenome-wide association studies (EWAS) have been completed with interesting novel candidate findings, e.g., related to the immune and endogene stress system [17, 18]. Few studies are available regarding the influence of life events (LE) on DNAm in PD with first interesting results. One study investigated recent negative LE and DNAm in the gene *Glutamate Decorboxylase* (*GAD1*) involved in GABA synthesis and showed overall lower DNAm levels, specifically for female PD patients [19]. Similar results have been reported for the *Monoamine Oxidase A* gene (*MAOA*) [20]. Finally, one study investigating a novel candidate gene for PD, *TMEM132D*, derived from a genome-wide association study (GWAS) [21], showed a positive correlation of DNAm with positive LE [22]. However, no EWAS on LE and PD has been available yet.

PD often co-occurs with major depression (MDD), the lifetime comorbidity rates are estimated at 50–80% [23, 24]. PD and MDD are characterized as stress-related disorders as stressful life events are important contributing factors to the etiology and clinical course in both disorders. Generally, it is unclear which molecular pathways induced by external stress are common between PD and MDD and which might be specific for to the phenotypic difference. Therefore, in addition to the first evidence on shared and distinct genetic basis between PD and MDD from cross-disorder GWAS data [25], stress-induced DNAm changes might be the missing link to explain common and disorder-specific biological patterns. To date, one study by Zannas et al. in MDD analyzed EWAS data and stress on age prediction and epigenetic clocks. The results showed that cumulative life stress was linked to accelerated epigenetic age and that this effect could be driven by glucocorticoid induced DNAm [26].

In the present study, we investigated the influence of LE on epigenome-wide DNAm in two PD cohorts (183 patients with PD in total) as well as the interaction between PD status and LE. As the stressfulness of LE may vary between subjects, we additionally evaluated the perceived burden related to the reported LE. Given the high comorbidity of PD with MDD, we additionally conducted the same analysis in a case sample of MDD including 102 patients in order to confer disease-specific and common DNAm changes in response to LE.

## Results

An overview of all performed analyses is given in Additional file 1: Fig. S1.

### EWAS on weighted stressful life events in panic disorder

First, we assessed associations of weighted stressful life events (wLE), weighted positive (wposLE) and weighted negative LE (wnegLE) with DNA methylation (DNAm) levels in the PD discovery (PDI) and replication (PDII) case samples (see Table 1) on an epigenome-wide scale. Afterward, we meta-analyzed the results from both cohorts. Manhattan- and QQ-plots for the individual EWAS in PDI and PDII as well as for the meta-analysis are depicted in Additional files 2–4: Figs. S2–S4.

**Table 1:** Demographics of included samples

	PD discovery (PDI)	PD replication (PDII)	MDD cases	*p*-value Chi-Square*	*p*-value ANOVA*
*n*	109	159	102		
Cases (%)	87 (79.81%)	96 (60.38%)	102 (100%)	< 0.01	NA
Controls (%)	22 (20.19%)	63 (39.62%)	NA	< 0.01	NA
Male (%)	44 (40.37%)	54 (33.96%)	64 (62.75%)	< 0.01	NA
Female (%)	65 (59.63%)	105 (66.04%)	38 (37.25%)	< 0.01	NA
Mean age (SD)	47.65 (9.65)	38.23 (10.27)	47.76 (13.63)	NA	< 0.01
Mean log wLE (SD) in cases	4.16 (0.61)	4.21 (0.67)	4.26 (0.61)	NA	0.59

^*^p-value for differences in means of quantitative variables are based on ANOVA, *p*-values for differences in proportions of categorical variables are based on Chi-Square-Test

wLE: weighted life events

**Fig. 1:**
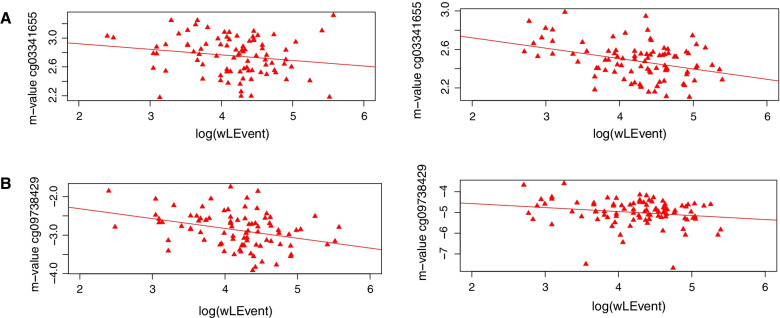
Scatterplots for top hits of meta-analysis on EWAS in PD discovery and PD replication sample. Scatter plot of *M*-value of cg03341655 (*y*-axis) and log(wLE) (*x*-axis) in PD discovery (left) and replication sample (right) (**A**). Scatter plot of *M*-value of cg09738429 (*y*-axis) and log(wLE) (*x*-axis) in PD discovery **(**left) and replication sample (right) (**B**)

While no result was significant at FDR of 5%, two CpG-sites represented with *p*-values below 1.0 × 10^−05^ (see Table 2 and Additional file 4: Fig. S4) in the meta-analysis of wLE: cg03341655 (see Fig. 1A), an exonic CpG in *Glucose-fructose oxidoreductase domain containing 2* (*GFOD2)* gene on chromosome 16, and cg09738429 (see Fig. 1B), located in an intergenic region on chromosome 12 between the *Solute carrier organic anion transporter family, member 1A2 (SLCO1A2,* 102 kb downstream) and in the next proximity of *Pyridine nucleotide-disulphide oxidoreductase domain 1* (*PYROXD1*, 87 bp upstream). For both CpG-sites in discovery as well as in the replication cohort, PD cases presented with higher levels of wLE and lower DNAm levels. Neither the top hit of the EWAS on PD nor the studied candidate genes in Iurato et al. [18] were associated with wLE.

**Table 2:** Top hits of meta-analysis of wLE in PD discovery (PDI) and replication sample (PDII)

CpG	beta_meta	p_meta	p_meta_corrected	beta_PDI	p_PDI	beta_PDII	p_PDII
cg09738429	− 0.2656	6.40 × 10^−06^	1.00	− 0.2798	2.36 × 10^−04^	− 0.2382	2.05 × 10^−02^
cg03341655	− 0.1077	8.14 × 10^−06^	1.00	− 0.0686	1.67 × 10^−01^	− 0.1201	3.98 × 10^−05^

beta_meta: effect-size estimate in meta-analysis

p_meta: nominal *p*-value from meta-analysis

p_meta_corrected: Benjamini–Hochberg FDR-corrected *p*-value from meta-analysis

beta_PDI: effect-size estimate in PDI

p_PDI: nominal *p*-value in PDI

beta_PDII: effect-size estimate in PDII

p_PDII: nominal *p*-value in PDII

The weighted LE score included positive as well as negative events but was mainly correlated with weighted negative LE (wnegLE, discovery sample PDI: Spearman’s *r* = 0.90, *p* = 1.88 × 10^−20^, replication sample PDII: Spearman’s *r* = 0.88, *p* = 1.26 × 10^−26^) and to a far lower extent with weighted positive LE (wposLE, PDI: Spearman’s *r* = 0.24, *p* = 0.083, PDII: Spearman’s *r* = 0.29, *p* = 0.01). Weighted positive and weighted negative LE were not correlated with each other (PDI: Spearman’s *r* = 0.05, *p* = 0.69, PDII: Spearman’s *r* = 0.06, *p* = 0.58). We therefore repeated the analysis on wposLE (see Additional files 5–7: Figs. S5–S7) and wnegLE separately (see Additional files 8–10: Figs. S8–S10). Again, no CpG-sites were significant at FDR of 5%. Seven CpG-sites presented with *p*-values below 1.0 × 10^−05^ in the meta-analysis of wposLE, 8 CpGs in the meta-analysis of wnegLE, no CpGs overlapped (see Additional file 11: Tables S1 and S2). Furthermore, no CpGs were significantly differentially associated with wposLE and wnegLE, i.e., showing different effect directions between positive and negative LE, with *p*-values below 1.0 × 10^−03^.

### Pathway enrichment analysis of top hits

Next, we performed a pathway enrichment analysis based on all genes mapping to CpG-sites associated with wLE with *p*-values < 0.01 in the meta-analysis (1995 CpG-sites mapping to 1742 unique genes). We used genes mapping to all CpG-sites included in the meta-analysis as background (424,763 CpG-sites mapping to unique 19,563 genes).

Genes mapping to the top hits were specifically expressed in brain (*p* = 7.73 × 10^−05^), followed by blood vessel, breast and Fallopian tube (see Additional file 11: Table S3). The enrichment for brain was mainly driven by CpG-sites which were higher methylated with higher wLE scores (872 hypermethylated CpGs-sites mapping to 787 unique genes, see Additional file 11: Table S4). CpG-sites which were lower methylated with higher wLE scores (1123 hypomethylated CpGs mapping to 1024 unique genes) did not show any tissue-specific enrichments (see Additional file 11: Table S5). A similar pattern arose for enrichment for GO biological processes: genes matching to top hits, regardless of direction, were significantly enriched for 247 GO biological processed terms including embryo (*p* = 1.24 × 10^−14^) and neuron development (*p* = 7.96 × 10^−09^, see Additional file 11: Table S6). These enrichments were again mainly driven by hypermethylated CpG-sites (enriched for 137 terms, see Additional file 11: Table S7; hypomethylated CpG-sites were enriched for only 52 terms, see Additional file 11: Table S8).

### DMR of weighted stressful life events

**Fig. 2:**
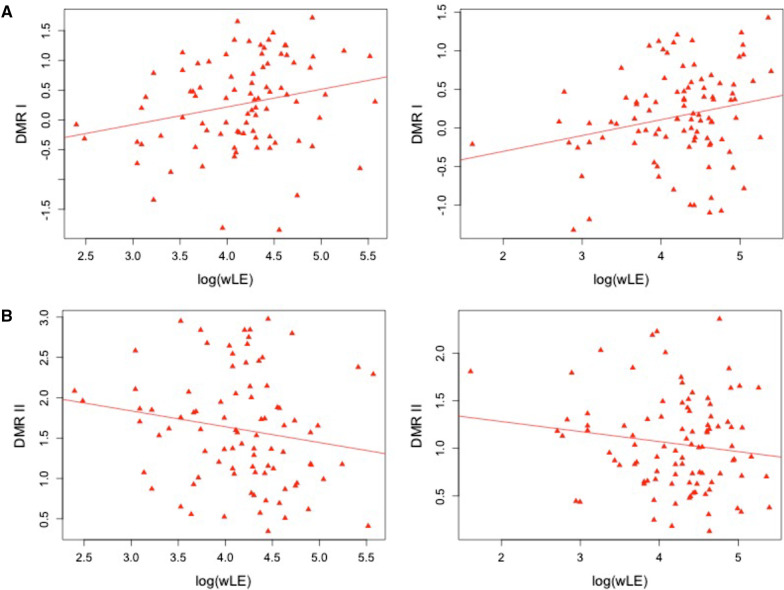
Scatterplots for top DMRs of meta-analysis on EWAS in PD discovery and PD replication sample. Scatter plot of mean *M*-value of DMR I at chr 10: 10,1282,726–101,282,884 (*y*-axis) and log( wLE) (*x*-axis) in PD discovery (left) and replication sample (right) (**A**). Scatter plot of mean *M*-value of DMR II at chr 18:72,837,531–72,837,701 (*y*-axis) and log(wLE) (*x*-axis) in PD discovery (left) and replication sample (right) (**D**)

Analysis on DMRs of wLE revealed two significant regions at FDR 5% (see Table 3): DMR I located in an intergenic region on chromosome 10: 10,1282,726–101,282,884 between *GOT1* (92 kb downstream) and *DQ372722* (3 kb upstream) and consisting of 4 CpG-sites (cg01987516, cg07044859, cg17888390 and cg23904955) where individuals with a higher score of wLE presented with higher DNAm levels (see Fig. 2A). Direction of effects was opposite for DMR II located in an intergenic region on chromosome 18: 72,837,531–72,837,701 between *ZNF407* (60 kb downstream) and *ZADH2* (72 kb upstream), consisting of 4 CpG-sites (cg04756515, cg14395744, cg18709881 and cg21894287, see Fig. 2B).

**Table 3:** Top hits of DMR-analysis of wLE in PD discovery (PDI) and replication sample (PDII)

CpGs included in DMR	Position (hg19)	beta_PDI	p_PDI	beta_PDII	beta_PDII	p_DMR	p_DMR_corrected
cg01987516, cg07044859, cg17888390, cg23904955	chr10: 10,1282,726–101,282,884	1.4077	1.14 × 10^−02^	1.0837	4.91 × 10^−03^	2.11 × 10^−11^	5.60 × 10^−08^
cg04756515, cg14395744, cg18709881, cg21894287	chr18: 72,837,531–72,837,701	− 1.008	5.49 × 10^−02^	− 0.7770	1.18 × 10^−02^	2.03 × 10^−08^	5.06 × 10^−05^

beta_PDI: effect-size estimate in PDI when taking the mean methylation M-values across all CpGs included in the DMR

p_PDI: nominal *p*-value in PDI when taking the mean methylation M-values across all CpGs included in the DMR

beta_PDII: effect-size estimate in PDII when taking the mean methylation M-values across all CpGs included in the DMR

p_PDII: nominal *p*-value in PDII when taking the mean methylation M-values across all CpGs included in the DMR

p_DMR: nominal *p*-value from DMR-analysis in comb-p on the meta-analysis of PDI and PDII

p_DMR_corrected: *p*-value from DMR-analysis in comb-p on the meta-analysis of PDI and PDII corrected for multiple testing across all tested regions

The DMR analysis was performed in comb-p. The effect sizes in PDI and PDII using the mean *M*-values are just displayed for illustration. Comb-p uses the meta-analysis *p*-values of the single CpGs directly

### Interaction of PD versus control status on wLE

Next, we investigated interaction effects of case–control status and wLE on DNAm, i.e., if the association of wLE on DNAm differed between PD cases and controls (see Additional files 12–14: Figs. S11–S13). We observed no significant interaction for single CpGs or for DMRs in the meta-analysis passing multiple testing correction. Seven CpGs presented with interaction *p*-values below 1.0 × 10^−05^ (see Additional file 11: Table S9). The strongest interaction was observed for *cg20941758*, an intronic CpG-site in *NKAIN1* on chromosome 1 (p_interaction_meta = 1.52 × 10^−06^, see Additional file 15: Fig. S14). While PD cases presented with higher DNAm levels with lower wLE, controls presented with higher DNAm levels with higher wLE.

### EWAS on weighted stressful life events in major depressive disorder and meta-analysis with panic disorder

Finally, we assessed associations of wLE with DNAm levels in the MDD case sample (see Table 1) on an epigenome-wide scale (see Additional file 16: Fig. S15). We observed no associations surviving multiple testing correction. The top hit was cg00769012, an intronic CpG-site in *SYNGR1* on chromosome 22 (*p* = 4.73 × 10^−05^) where wLE was associated with higher DNAm levels. This CpG was not associated in the PD samples (p_meta = 0.31). As the MDD-sample presented with low power by itself, we meta-analyzed it with the PD discovery and replication sample (see Additional file 17: Fig. S16). The same two CpG-sites that had already evolved in the PD only analyses, presented also here with meta *p*-values < 1.0 × 10^−05^: cg03341655 in *GFOD2,* here the *p*-value got more significant when adding the MDD cases (p_meta = 6.90 × 10^−06^) indicating a replication (in MDD cases: beta =  − 0.0554, *p* = 2.80 × 10^−01^, also see Additional file 18: Fig. S17). For cg09738429, adding the MDD cases increased the *p*-value (p_meta = 9.77 × 10^−06^). To further evaluate if associations of wLE with DNAm were consistent in PD as well as in MDD cases, we investigated if direction of effects were different between PD cohorts and the MDD cohort. Focusing on the top-hits (*p* < 1.0 × 10^−03^) from the PD meta-analysis, we observed that only 53 out of 136 top CpG-sites available in all three cohorts presented with consistent effect across all three cohorts indicating that some top hits seem be specific to PD cases.

## Discussion

The present study is the first to investigate the relationship of emotionally weighted life events on epigenome-wide DNAm in PD as diagnostic phenotype and its boundary to depression. No epigenome-wide significant results could be discerned in the PD cases meta-analysis; however, two CpG-sites presented with *p*-values below 1.0 × 10^−05^: cg09738429 (*p* = 6.40 × 10^−06^, located in an intergenic shore region in the next proximity of *PYROXD1*) and cg03341655 (*p* =  = 8.14 × 10^−06^, located in the exonic region of *GFOD2*). *PYROXD1* is involved in the response to oxidative stress [27]. Recent studies report higher DNAm in this gene in acute coronary syndrome and brain white matter lesions in older populations [28, 29]. Furthermore, a microarray-based post mortem analysis in human dorsal raphe nucleus tissue, a brain region pathophysiologically involved in serotonergic neurotransmission in MDD, showed a significant upregulation of the *PYROXD1* transcript in MDD cases vs. controls, corresponding to higher protein production related to MDD [30]. In our study, high wLE levels were associated with reduced DNAm suggesting higher gene expression, although we are not aware of any data evaluating the functional relevance of cg09738429 on gene regulation. Therefore, from this first results we can only speculate that high life time emotional stress could affect regulation of *PYROXD1* through DNAm. Additionally, positive EWAS associations at several CpG-sites in *PYROXD1* are available for adult smoking and maternal smoking in pregnancy [31–33]. Interestingly in relation to life stress, several investigations revealed also significant contributions of CpG-sites in *PYROXD1* as epigenetic markers for aging [34, 35].

Cg03341655, located in *GFOD2*, was the second top hit*.* Only for this CpG the significance level increased when patients with MDD were added to the meta-analysis suggesting a diagnosis independent effect of wLE on DNAm. *GFOD2* is highly expressed in the brain, predominantly in the cerebellum and cerebral cortex, lower expression has been shown in the hypothalamus and pituitary gland. However, its functional implication in psychiatric phenotypes, and specifically in anxiety, remains unclear. SNPs in the *GFOD2* have been associated with schizophrenia related phenotypes [36] and a recent study using a zebrafish model revealed its implication in the developing and adult brain as well as *GFOD2* expression in a subset of inhibitory GABA-neurons [37]. Variants in *GFOD2* have also been linked to the metabolic system and coronary disease, e.g., levels of circulating lipid levels and differential response to cholesterol-lowering diet [38]. One study showed negative correlation of DNAm of cg03341655 in the subcutaneous adipose tissue in response to high saturated fatty acids diet [39]. In fact, both, PD and MDD, as well as chronic stress are related to higher risk of metabolic syndromes and cardiovascular morbidity as has been shown in multiple studies [40–42]. Further EWAS have reported DNAm changes at different CpG-sites in *GFOD2* in relation to childhood abuse [43], maternal alcohol consumption and offspring cord blood methylation [44] and all-cause mortality in monozygotic twins [45]. These epigenetic studies suggest the relation of *GFOD2* DNAm status to a broad spectrum of stressful environmental conditions across life span.

One hypothesis for metabolic changes in anxiety and depression is the pronounced stimulation and putative hyperactivation of the endogenous stress system by life events [13, 46]. In MDD, this has also been related to higher age acceleration determined by epigenetic age markers [26]. However, no studies are available whether high levels of cortisol could lead to DNAm changes in *GFOD2*. One investigation in mice reports DNAm changes of the *Gfod2* gene in oocytes exposed to superovulation showing that gonadotropin hormones, at least in a model with very high dosages, can induce DNAm changes in the respective gene [47]. However, the functional consequences of DNAm changes in *GFOD2* are widely unknown und further experiments involving neuroendocrine and metabolic measurements in reaction to stress are needed.

In summary, the analyses of differential DNAm in PD and MDD in response to wLE points to molecular targets implicated in the metabolic system, although the knowledge of the exact role of these first candidates remain limited. Nevertheless, pathway enrichment analysis primarily maps the top hits to genes expressed in the brain and secondly to blood vessels, suggesting that the present results map DNAm functionally involved in the transmission of stress effects also to brain systems.

The top hits from the analysis restricted to negative life events highly correlated with findings using the total life event composite score. When looking at positive life events, no previous EWAS data are available for our specific hits. However, EWAS have been published for other CpG-sites in the corresponding genes, presenting different directions of DNAm in association with disease phenotypes (e.g., for *TNXB* for maternal smoking and childhood abuse: [43, 48]). In the PD case–control interaction EWAS analysis on wLE, the best nominal association was located in the gene *NKAIN1,* showing opposite direction of DNAm in cases and controls with increasing values of wLE. *NKAIN1* is expressed in the brain, specifically in hippocampus, bus also in musculature and endocrine gland [49]. Variants in this gene have been associated with alcohol dependence [50] and autism spectrum disorder [51]. In the present study, only PD patients without history of alcohol dependence were included, suggesting that the differential DNAm methylation found here is not attributed to this phenotype. Indeed, the possible implication of *NKAIN1* in life stress related DNAm in PD as diagnosis remains unclear so far and further studies are needed to clarify how this gene is involved in the common and distinct biological pathways crossing PD, MDD and alcohol phenotypes.

We also conductd a DMR-analysis as this reveals more robust associations on regional clusters as compared to association analysis on single CpGs. The DMR analysis in PD patients resulted in two genomic regions significantly associated with differential methylation and wLE. The first DMR containing 4 CpGs (cg01987516, cg07044859, cg17888390 and cg23904955) is located on chr10 in an intergenic region between the genes *GOT1* and *DQ372722*. Higher DNAm of cg01987516 has been previously related to maternal anxiety in umbilical cord blood [52]. Furthermore, cg23904955 has been negatively correlated with ethanol consumption per day over the course of a year during the blood sample collection in a European population [53]. In addition, some CpGs have been positively correlated with insulin sensitivity and BMI in early childhood [54] and negatively corelated with smoking [31], which could be referred to the general field of metabolic health. The second significant DMR containing four CpGs on chromosome 18 is located in an intergenic region between the genes *ZNF407* and *ZADH2*. So far, no DNAm data on psychiatric phenotypes have been linked to this specific region. However, genetic variants as well as CpGs in both proximal genes have been associated with various phenotypes related to neuropsychiatric diseases. Genetic and methylation studies revealed associations in *ZNF407* with neurodevelopmental disorders, schizophrenia as well as Gulf War illness [55, 56]. One study points to the putative role of *ZNF407* in the regulation of insulin-stimulated glucose uptake [57]. Differential DNAm in *ZADH2* has been shown for suicidal attempts in schizophrenia [58], memory performance in Alzheimers disease [59], but also inflammatory phenotypes [60].

The present study offers the first results of DNAm in association with cumulative life events in PD and its boundary to depression. The interpretation of the results is limited by the moderate number of the included individuals. We study DNAm in blood and our results cannot be directly related to DNAm in brain. Investigations of tissue’s average methylation for all CpGs between blood and brain show divergent correlation values, these values range from levels lower than 0.1 up to the overall blood–brain DNAm correlation of around 0.8 in a recent study [61]. Furthermore, PD patients were free of substance use disorders and were not medicated at the time of inclusion, but this does not account for putative psychiatric/non-psychiatric medication in the past. In contrast, MDD patients were medicated which could have influences on DNAm levels as has been shown by Barbu et al. [62]. Other factors not included in our analysis, such as childhood adversity, perinatal factors and further environmental influences might bias our analysis. Furthermore, the included PD cohorts cannot be treated as totally independent. The replication cohort is different in time of recruitment and time of DNAm measurement, however, similar in genetic architecture, assessment strategy and diagnostic evaluation given that both PD cohorts were recruited in the same center. It should also be noted that our study was performed on 450K arrays and hence, we could have missed potentially significant sites which are not covered on this array. Therefore, replication of our results in completely independent cohorts as well evaluation of CpGs covered by the EPIC array is necessary. However, by using a meta-analytic approach and adding MDD cases we aimed to provide a robust level of replication. Furthermore, we thoroughly corrected for smoking, cell types, age as well as sex, which are important confounding variables.

## Conclusions

In summary, this first DNAm analysis in PD reveal first evidence of small but significant DNAm changes in PD in association with cumulative stress-weighted life events. DMR analyses in PD rendered more disease-specific DNAm changes in relation to wLE in comparison to the EWAS, as seen by the additional analysis with MDD. Most of the top associated CpGs were located in genes implicated in metabolic processes supporting the hypothesis that environmental stress contributes to health damaging changes by affecting a broad spectrum of systems in the body which might contribute to age acceleration, as shown for affective disorders [26, 63]. The specificity of the DNAm results has to be replicated in independent samples providing measurement of endocrine, vascular and cardiac function in combination with DNAm and life stress in PD.

## Methods

### Study samples

#### Panic disorder (PD) discovery and replication sample

The PD discovery and replication sample are the same cohorts which were used in Iurato et al. [18] and were named as discovery and replication as they form two time-independent batches.

PD patients included in the discovery (*n* = 87) and replication sample (*n* = 96) were recruited in the anxiety disorders outpatient unit at the MPIP in Munich [21], (see Table 1 for demographic details). PD was the primary diagnosis ascertained by trained psychiatrists according to the Diagnostic and Statistical Manual of Mental Disorders (DSM)-IV criteria. Mild secondary depression was allowed. All patients underwent the Structured Clinical Interviews for DSM-IV (SCID I and II) [64]. PD due to a medical or neurological condition or the presence of a comorbid Axis II disorder was an exclusion criterion.

Control subjects were recruited from a Munich-based community sample and screened for the absence of axis I psychiatric disorders with the SCID [64]. Controls were age- and sex-matched with patients. To reduce confounding due to possible effects of drug treatment, both patients and controls were free of psychotropic medication for at least 4 weeks before the blood draw. All subjects were Caucasian and provided written informed consent. The Ethics Committee of the Ludwig Maximilians University, Munich, Germany, in accordance with the Declaration of Helsinki approved all procedures, Project number 318/00.

#### Major depressive disorder (MDD) sample

An independent sample of 102 depressed patients with information available on stressful life events was recruited at the MPIP. Recruitment strategies and detailed characterization of participants for the whole sample have been described elsewhere [65, 66]. In short, the diagnosis was ascertained by trained psychiatrists according to the DSM-IV criteria. Exclusion criteria were the presence of alcohol or substance abuse or dependence, comorbid somatization disorder, and depressive disorders owing to general medical or neurologic conditions. All patients were medicated. The study was approved by the local ethics committee and all individuals gave written informed consent.

### Stressful life events (SLE)

Life events (LE) were assessed using the “Event List” [67], which is a German adaptation of the Social Readjustment Scale by Holmes et al. [68]. The event list includes 37 items assessing the occurrence and frequency (once, twice, several times) of typical life events including marriage, separation, change in life standards and habits, as well as death of close relatives and friends. Each item was additionally rated with respect to personal valency (very positive to very negative) and burden (not burdensome at all to extremely burdensome) on a 5-item Likert scale. From all 37 items a total life events score and a stress-weighted total life event score (wLE) were calculated reflecting the overall frequency of all life events and the overall life events frequency weighted by the individual burden score, respectively. In addition, items were categorized according to the individual valency score as positive or negative; the average number of positive life event items was 5.47 (ranging from 0 to 15), while the average number of negative life event items was 6.09 (ranging from 0 to 20). From these data, the numbers of positive and negative life events were calculated. In addition, weighted negative life events were obtained by weighting the reported event number with the individual burden scores (wnegLE), while weighted positive life events were derived by weighting the reported event number with the inverted individual burden scores (wposLE), reflecting positive life events weighted by individual relief.

To reduce the influence of extreme observations on the model parameters we applied a logarithmic transformation to wLE in the association analysis.

#### DNA methylation (DNAm) in PD sample

The pre-preprocessing is described in detail in [18]. Briefly, genomic DNA was extracted from peripheral blood and bisulfite converted DNA methylation levels were assessed for > 480,000 CpG sites using the Illumina HumanMethylation450 BeadChip array. The Bioconductor R package *minfi* [69] was used for the quality control of DNAm data. Failed probes based on a detection *P*-value larger than 0.01 in > 50% of the samples as well and non-specific binding probes [70] and probes on X and Y chromosome were removed. We also excluded probes if single nucleotide polymorphisms (SNPs) were documented in the interval for which the Illumina probe is designed to hybridize. Probes located close (10 bp from query site) to a SNP which had a minor allele frequency of ≥ 0.05, as reported in the 1000 Genomes Project, were also removed. The data were then normalized with functional normalization [71]. Batch effects were identified using the Empirical Bayes’ method *ComBat*. Batch corrected *M*-values after *ComBat* [72] were used for all further statistical analyses. Cell-type proportions were estimated from DNA methylation levels using the Houseman algorithm [73]. Furthermore, we derived smoking scores based on [74].

#### DNAm in MDD sample

Pre-processing is described in detail in [65]. Genomic DNA was extracted from whole blood and DNA methylation levels were assessed for > 480,000 CpG sites using the Illumina HumanMethylation450 BeadChip arrays. All methylation probes have been subjected to an extensive quality control including filtering by low p-detection value, functional normalization and batch correction with ComBat. Cellular composition was estimated by using CellCode [75]. Furthermore, we derived smoking scores based on Zeilinger et al. [74].

### Statistical analyses

#### Epigenome-wide association analysis (EWAS) with wLE

First, within each cohort separately, association between log(wLE) and DNAm levels were assessed using linear regression models in R. The analysis was repeated on log(wposLE) and log(wnegLE). *M*-values of each CpG-site were used as dependent variable, log(wLE), log(wposLE) or log(wnegLE) respectively as independent variables. Age, sex, estimated cell type proportions as well as smoking score were used as covariates. For this analysis, only PD cases or MDD cases were included.

#### Case–control interaction with wLE

Within each PD cohort separately, we also tested for interaction between log(wLE), log(wposLE) and log (wnegLE) and PD case–control status on DNAm levels using linear regression models in R. *M*-values of each CpG-site were used as dependent variable, log(wLE) × PD case–control status as independent variable. The interaction model included the main effects of log(wLE) and PD case–control status. Age, sex, estimated cell type proportions as well as smoking score were used as covariates.

#### Meta-analysis

As PD discovery and replication were assessed timely independent of each other on the methylation arrays and hence can be considered as two independent batches, we meta-analysed the association results combining PD discovery and replication samples, a strategy which was also chosen in the original publication by Iurato et al. [18] who studied PD case–control effects on DNAm in these two cohorts. Meta-analysis combining PD discovery and replication samples as well as the MDD cohort (for the EWAS on wLE) was performed using PLINK v1.9. [76]. In PLINK, we used the meta-analysis command and report *p*-values from a fixed-effects meta-analysis. Overall, 424,763 CpGs were available in both PD cohorts and 308,360 CpGs across all three cohorts.

#### Manhattan- and QQ-plots

Manhattan- and QQ-plots were generated using the R-package *qqman.* Lambda-values were calculated using the R-package *QCEWAS.*

#### Differential methylation regions (DMRs)

In order to identify clusters of association results in the EWAS, we performed DMR analysis on the meta-analysis results from both PD samples based on the input of individual *p*-values of at least 5.0 × 10^−05^ and within 500 bp using comb-P [77].

#### Pathway enrichment

We used FUMA v1.3.6a [78], specifically the GENE2FUNC option, to test top hits for pathway enrichment. First, all CpG-site included in the PD meta-analysis were annotated to the nearest gene using the *matchGenes* function in the R-package *bumphunter* [79]. These 424,763 CpG-sites matched to 19,563 unique genes. This set was used as background. Next, we used all CpG-sites associated with a *p*-value < 0.01 in the meta-analysis of the PD case only analysis. These 1995 CpG-sites mapped to 1743 unique genes. This gene set was used as input set. These two genes sets were provided to the GENE2FUNC which runs Fisher-tests for enrichment of pathways, tissue specific genes in GTEx v8 [80] and genes identified in several GWAS. The FDR cut-off was set to 5% and a minimal overlap of 10 genes between gene-sets to be present.

#### Multiple testing correction

All association results were corrected for multiple testing at and false-discovery-rate (FDR) of 5% using the method of Benjamini and Hochberg [81].

## Supplementary Information

**Additional file 1: Figure S1**. Overview of conducted analyses.

**Additional file 2: Figure S2**. Manhattan plot for EWAS of wLE in PDI cases. Chromosomal position is depicted on the *x*-axis, −log10(*p*-value) on the *y*-axis. The blue line indicates nominal *p*-values < 1.0 × 10^−05^ (A). QQ-plot for EWAS of wLE in PDI cases depicting expected −log10(*p*-values) versus observed −log10(*p*-values). The lambda-value is 0.83 (B).

**Additional file 3: Figure S3**. Manhattan plot for EWAS of wLE in PDII cases. Chromosomal position is depicted on the *x*-axis, −log10(*p*-value) on the *y*-axis. The blue line indicates nominal *p*-values< 1.0 × 10^−05^ (A). QQ-plot for EWAS of wLE in PDII cases depicting expected −log10(*p*-values) versus observed −log10(*p*-values). The lambda-value is 0.91 (B).

**Additional file 4: Figure S4**. Manhattan plot for meta-analysis of EWAS of wLE in PDI cases and PDII cases. Chromosomal position is depicted on the *x*-axis, −log10(*p*-value) on the *y*-axis. The blue line indicates nominal *p*-values < 1.0 × 10^−05^ (A). QQ-plot for meta-analysis of EWAS of wLE in PDI cases and PDII cases depicting expected −log10(*p*-values) versus observed −log10(*p*-values). The lambda-value is 0.86 (B).

**Additional file 5: Figure S5**. Manhattan plot for EWAS of wposLE in PDI cases. Chromosomal position is depicted on the *x*-axis, −log10(*p*-value) on the *y*-axis. The blue line indicates nominal *p*-values < 1.0 × 10^−05^ (A). QQ-plot for EWAS of wposLE in PDI cases depicting expected −log10(*p*-values) versus observed −log10(*p*-values). The lambda-value is 0.92 (B).

**Additional file 6: Figure S6**. Manhattan plot for EWAS of wposLE in PDII cases. Chromosomal position is depicted on the *x*-axis, −log10(*p*-value) on the *y*-axis. The blue line indicates nominal *p*-values < 1.0 × 10^−05^ (A). QQ-plot for EWAS of wposLE in PDII cases depicting expected −log10(*p*-values) versus observed −log10(*p*-values). The lambda-value is 1.00 (B).

**Additional file 7: Figure S7**. Manhattan plot for meta-analysis of EWAS of wposLE in PDI cases and PDII cases. Chromosomal position is depicted on the *x*-axis, −log10(*p*-value) on the *y*-axis. The blue line indicates nominal *p*-values < 1.0 × 10^−05^ (A). QQ-plot for meta-analysis of EWAS of wposLE in PDI cases and PDII cases depicting expected −log10(*p*-values) versus observed −log10(*p*-values). The lambda-value is 1.00 (B).

**Additional file 8: Figure S8**. Manhattan plot for EWAS of wnegLE in PDI cases. Chromosomal position is depicted on the *x*-axis, −log10(*p*-value) on the *y*-axis. The blue line indicates nominal *p*-values < 1.0 × 10^−05^ (A). QQ-plot for EWAS of wnegLE in PDI cases depicting expected −log10(*p*-values) versus observed −log10(*p*-values). The lambda-value is 0.91 (B).

**Additional file 9: Figure S9**. Manhattan plot for EWAS of wnegLE in PDII cases. Chromosomal position is depicted on the *x*-axis, −log10(*p*-value) on the *y*-axis. The blue line indicates nominal *p*-values < 1.0× 10^−05^ (**A**). QQ-plot for EWAS of wnegLE in PDII cases depicting expected −log10(*p*-values) versus observed −log10(*p*-values). The lambda-value is 0.94 (**B**).

**Additional file 10: Figure S10**. Manhattan plot for meta-analysis of EWAS of wnegLE in PDI cases and PDII cases. Chromosomal position is depicted on the *x*-axis, −log10(*p*-value) on the *y*-axis. The blue line indicates nominal *p*-values < 1.0 × 10^−05^ (A). QQ-plot for meta-analysis of EWAS of wnegLE in PDI cases and PDII cases depicting expected −log10(*p*-values) versus observed −log10(*p*-values). The lambda-value is 0.95 (B).

**Additional file 11: Table S1**. CpGs associated with weighted positive life-events in the meta-analysis of PD discovery and replication cases at *p* < 1.0x10^−05^. **Table S2**. CpGs associated with weighted negative life-events in the meta-analysis of PD discovery and replication cases at *p* < 1.0x10^−05^. **Table S3**. Enrichment analysis of top-hits in the meta-analysis of weighted life events of PD discovery and replication cases with regards to tissue specificity in GTEx. **Table S4**. Enrichment analysis of hypermethylated top-hits in the meta-analysis of weighted life events of PD discovery and replication cases with regards to tissue specificity in GTEx. **Table S5**. Enrichment analysis of hypomethylated top-hits in the meta-analysis of weighted life events of PD discovery and replication cases with regards to tissue specificity in GTEx. **Table S6**. Enrichment analysis of top-hits in the meta-analysis of weighted life events in PD discovery and replication caess with regards to GO biological processes. **Table S7**. Enrichment analysis of hypermethylated top-hits in the meta-analysis of weighted life events in PD discovery and replication caess with regards to GO biological processes. **Table S8**. Enrichment analysis of hypomethylated top-hits in the meta-analysis of weighted life events of PD discovery and replication cases with regards to tissue specificity. **Table S9**. CpGs associated with weighted life-events x case-control status in the meta-analysis of PD discovery and replication sample at *p* < 1.0 x10^−05^.

**Additional file 12: Figure S11**. Manhattan plot for EWAS of wLE x case-control-status in PDI. Chromosomal position is depicted on the *x*-axis, −log10(*p*-value) on the *y*-axis. The blue line indicates nominal *p*-values <1.0 × 10^−05^ (A). QQ-plot for EWAS of wLE x case-control-status in PDI depicting expected −log10(*p*-values) versus observed −log10(*p*-values). The lambda-value is 1.02 (B).

**Additional file 13: Figure S12**. Manhattan plot for EWAS of wLE x case-control-status in PDII. Chromosomal position is depicted on the *x*-axis, −log10(*p*-value) on the *y*-axis. The blue line indicates nominal *p*-values < 1.0 × 10^−05^ (A). QQ-plot for EWAS of wLE x case-control-status in PDII depicting expected −log10(*p*-values) versus observed −log10(*p*-values). The lambda-value is 0.99 (B).

**Additional file 14: Figure S13**. Manhattan plot for meta-analysis of EWAS of wLE x case-control-status in PDI and PDII. Chromosomal position is depicted on the *x*-axis, −log10(*p*-value) on the *y*-axis. The blue line indicates nominal *p*-values < 1.0 × 10^−05^ (A). QQ-plot for meta-analysis of EWAS of of wLE x case-control-status in PDI and PDII depicting expected −log10(*p*-values) versus observed −log10(*p*-values). The lambda-value is 1.02 (B).

**Additional file 15: Figure S14**. Scatterplots in PDI (above) and PDII (below) forF wigLuE on DNAm of cg20941758. The *x*-axis denotes log(wLE), the *y*-axis denotes *M*-value of cg20941758, PD cases are depicted in red, controls in green. The red line indicates the regression line in PD cases, the green line in controls.

**Additional file 16: Figure S15**. Manhattan plot for EWAS of wLE in MDD cases. Chromosomal position is depicted on the *x*-axis, −log10(*p*-value) on the *y*-axis. The blue line indicates nominal *p*-values < 1.0 × 10^−05^ (A). QQ-plot for EWAS of wLE in MDD cases depicting expected −log10(*p*-values) versus observed −log10(*p*-values). The lambda-value is 0.79 (B).

**Additional file 17: Figure S16**. Manhattan plot for meta-analysis of EWAS of wLE in PDI cases, PDII cases and MDD cases. Chromosomal position is depicted on the *x*-axis, −log10(*p*-value) on the *y*-axis. The blue line indicates nominal *p*-values <1.0 × 10^−05^ (A). QQ-plot for meta-analysis of EWAS of wLE in PDI cases, PDII cases and MDD cases cases depicting expected −log10(*p*-values) versus observed −log10(*p*-values). The lambda-value is 0.85 (B).

**Additional file 18: Figure S17**. Scatterplots in PD discovery (above), PD replication (middle) and MDD sample (below) for wLE on DNAm of cg03341655. The *x*-axis denotes log(wLE), the *y*-axis denotes *M*-value of cg03341655. The black line indicates the regression line.

## Data Availability

DNA methylation levels as well PD and weighted life events scores for PDI and PDII  have been deposited in NCBI's Gene Expression Omnibus and are accessible through GEO Series accession number GSE201016 (https://www.ncbi.nlm.nih.gov/geo/query/acc.cgi?acc=GSE201016).
