# Supplementary figures and images for: Effects of stressful life-events on DNA methylation in panic disorder and major depressive disorder
Source: Clin Epigenetics. 2022 Apr 27;14:55. doi: 10.1186/s13148-022-01274-y (PMC9047302; doi:10.1186/s13148-022-01274-y)

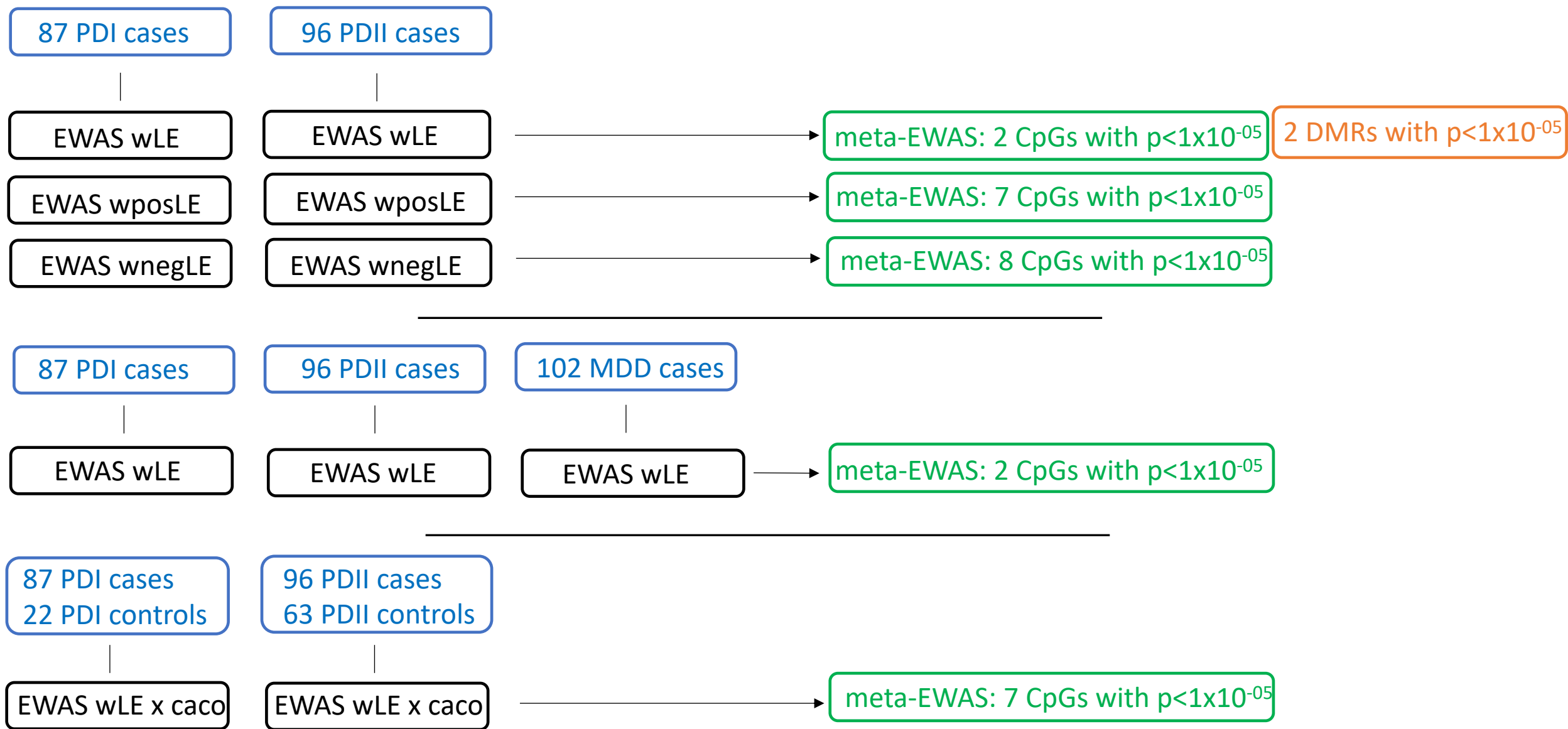

**Figure S1:** Overview of conducted analyses

Supplement: Supplementary file 1 — Additional file 1: Figure S1. Overview of conducted analyses. [file 13148_2022_1274_MOESM1_ESM.pdf]
