# Supplementary material for: Effects of stressful life-events on DNA methylation in panic disorder and major depressive disorder
Source: Clin Epigenetics. 2022 Apr 27;14:55. doi: 10.1186/s13148-022-01274-y (PMC9047302; doi:10.1186/s13148-022-01274-y)
Supplement: Supplementary file 5 — Additional file 5: Figure S5. Manhattan plot for EWAS of wposLE in PDI cases. Chromosomal position is depicted on the x-axis, −log10(p-value) on the y-axis. The blue line indicates nominal p-values < 1.0 × 10^−05^ (A). QQ-plot for EWAS of wposLE in PDI cases depicting expected −log10(p-values) versus observed −log10(p-values). The lambda-value is 0.92 (B). [file 13148_2022_1274_MOESM5_ESM.pdf]

A B

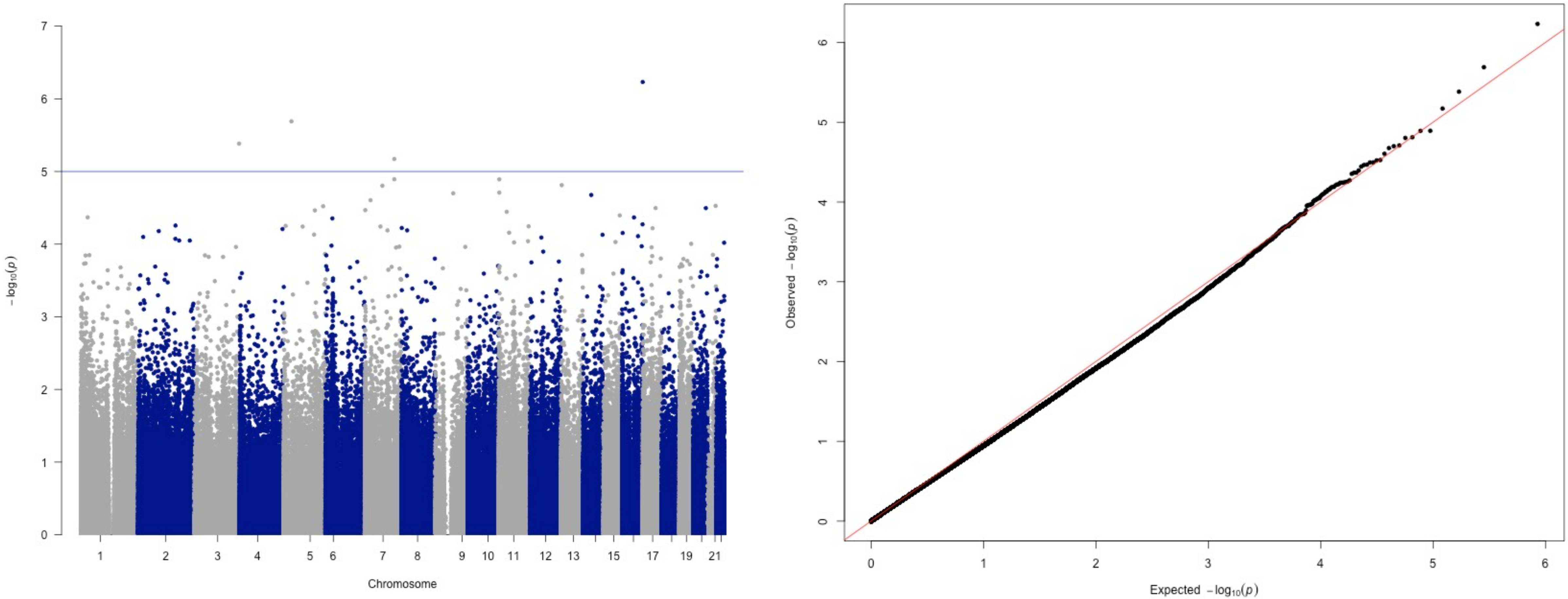

Figure S5: Manhattan plot for EWAS of wposLE in PDI cases. Chromosomal position is depicted on the x-axis, -log10(p-value) on the y-axis. The blue line indicates nominal p-values<1x10^{-05} (**A**). QQ-plot for EWAS of wposLE in PDI cases depicting expected –log10(p-values) versus observed –log10(p-values). The lambda-value is 0.92 (**B**).
