# Supplementary material for: Effects of stressful life-events on DNA methylation in panic disorder and major depressive disorder
Source: Clin Epigenetics. 2022 Apr 27;14:55. doi: 10.1186/s13148-022-01274-y (PMC9047302; doi:10.1186/s13148-022-01274-y)
Supplement: Supplementary file 15 — Additional file 15: Figure S14. Scatterplots in PDI (above) and PDII (below) forF wigLuE on DNAm of cg20941758. The x-axis denotes log(wLE), the y-axis denotes M-value of cg20941758, PD cases are depicted in red, controls in green. The red line indicates the regression line in PD cases, the green line in controls. [file 13148_2022_1274_MOESM15_ESM.pdf]

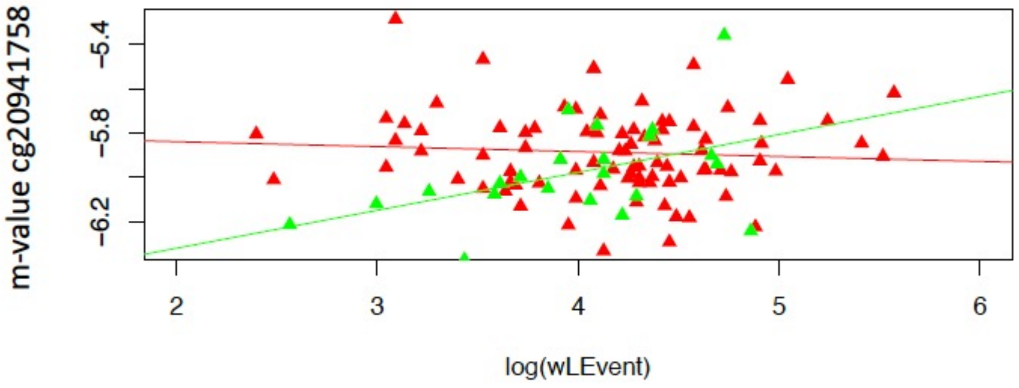

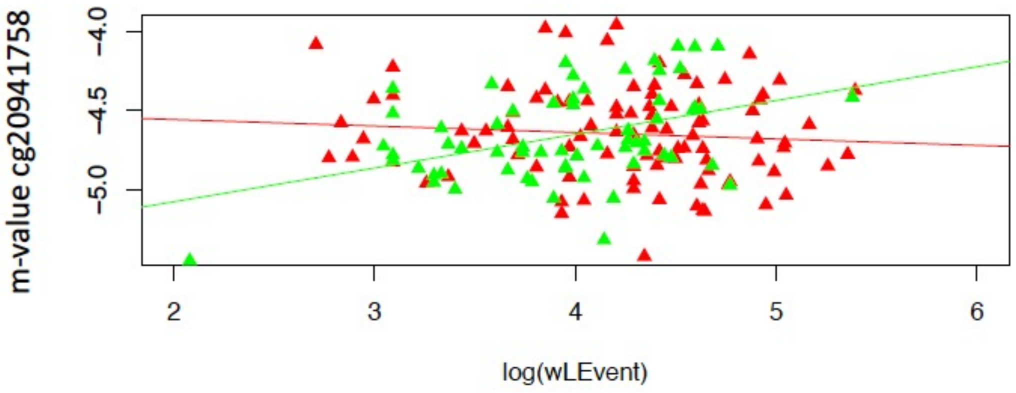

Figure S14: Scatterplots in PDI (above) and PDII (below) for wLE on DNAm of cg20941758. The x-axis denotes log(wLE), the y-axis denotes M-value of cg20941758, PD cases are depicted in red, controls in green. The red line indicates the regression line in PD cases, the green line in controls.
