# Supplementary material for: Effects of stressful life-events on DNA methylation in panic disorder and major depressive disorder
Source: Clin Epigenetics. 2022 Apr 27;14:55. doi: 10.1186/s13148-022-01274-y (PMC9047302; doi:10.1186/s13148-022-01274-y)
Supplement: Supplementary file 18 — Additional file 18: Figure S17. Scatterplots in PD discovery (above), PD replication (middle) and MDD sample (below) for wLE on DNAm of cg03341655. The x-axis denotes log(wLE), the y-axis denotes M-value of cg03341655. The black line indicates the regression line. [file 13148_2022_1274_MOESM18_ESM.pdf]

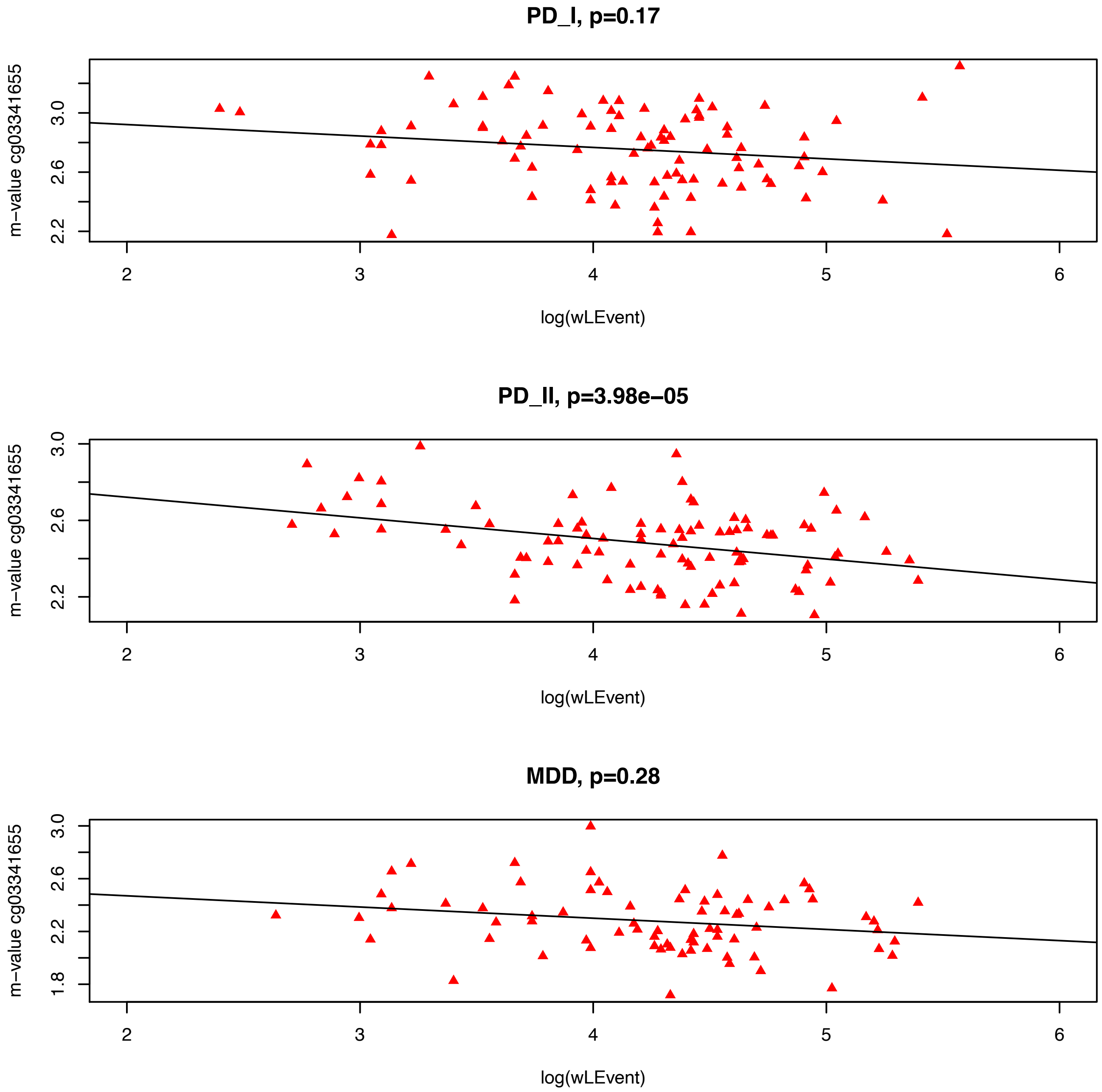

Figure S17: Scatterplots in PD discovery (above), PD replication (middle) and MDD sample (below) for wLE on DNAm of cg03341655. The x-axis denotes log(wLE), the y-axis denotes M-value of cg03341655. The black line indicates the regression line.
